# Clebopride stimulates 5-HT_4_-serotonin receptors in the human atrium

**DOI:** 10.1007/s00210-025-04075-1

**Published:** 2025-03-25

**Authors:** Lina Maria Rayo Abella, Joachim Neumann, Britt Hofmann, Uwe Kirchhefer, Ulrich Gergs

**Affiliations:** 1 Medical Faculty, Institute for Pharmacology and Toxicology, Martin Luther University Halle-Wittenberg, Magdeburger Straße 4, D-06112 Halle (Saale), Germany; 2 Medical Faculty, Institute for Pharmacology and Toxicology, University Münster, Münster, Germany; 3 Department of Cardiac Surgery, Mid-German Heart Centre, University Hospital Halle, Halle (Saale), Germany

**Keywords:** Clebopride, 5-HT_4_-Serotonin receptors, Human atrium, Transgenic mice

## Abstract

Clebopride resembles in its structural formula metoclopramide. Clebopride, an approved drug, is used to treat gastrointestinal diseases. Here, we tested the hypothesis that clebopride like metoclopramide acts as a partial agonist at human cardiac 5-HT_4_-serotonin-receptors. Clebopride enhanced the force of contraction (FOC) in isolated, electrically stimulated (1 Hz) left atrial preparations (LA) from transgenic mice with cardiac specific overexpression of the human 5-HT_4_-serotonin receptors (5-HT_4_-TG). Subsequently applied GR125487 (1 µM), a specific 5-HT_4_-serotonin-receptor antagonist, diminished this positive inotropic effect (PIE) of clebopride in LA from 5-HT_4_-TG. Clebopride failed to heighten FOC in LA from littermate wild-type mouse hearts (WT). Clebopride augmented the beating rate in isolated right atrial preparations (RA) from 5-HT_4_-TG but unable to do so in RA from WT. Clebopride alone (up to 10 µM) failed to augment FOC in isolated electrically stimulated (1Hz) human right atrial preparations (HAP) obtained during open heart surgery from adult patients with severe coronary heart disease. Interestingly, in the presence of the phosphodiesterase III inhibitor cilostamide, clebopride heightened FOC in HAP. GR125487 attenuated this PIE in HAP. Furthermore, when 1 µM serotonin had raised FOC in HAP, additionally applied 10 µM clebopride diminished FOC in HAP. We conclude that clebopride can act as an agonist and as an antagonist at 5-HT_4_-serotonin receptors in the human atrium.

## Introduction

Clebopride is an approved anti-dopaminergic gastrointestinal prokinetic drug. Moreover, ligand binding studies have shown that clebopride binds to 5-HT_4_-serotonin receptors and binds to D_2_-dopamine receptors (Masso and Roberts 1980, De Maeyer et al. 2008, Tack 2011, Tack et al. 2012). Thus, clebopride has prokinetic effects possibly via agonism at 5-HT_4_-aswathreceptors and/or possibly via antagonism at D_2_-dopamine receptors in the gut. Additionally, clebopride has anti-emetic effects probably via antagonism at the brain D_2_-dopamine receptors and possibly through antagonism at a brain 5-HT_3_-serotonin receptor (Duarte et al. 1985, Tack et al. 2012). Clebopride ranked best in its efficacy in a recent meta-analysis to improve the symptoms of gastroparesis (Bassotti et al. 2022, Abell et al. 2023, Ingrosso et al. 2023). Gastroparesis is a complication of, e.g. diabetes mellitus (Aswath et al. 2023). Clebopride is not available in many countries, including the USA and Germany. However, clebopride was developed in Spain, and therefore, clebopride is approved in Spain, Portugal, Italy, and some countries in South America and Asia (Reis-Carneiro et al. 2023). Hence, clebopride is clinically relevant in some countries, and therefore, we tried here to understand its conceivable but unknown cardiac (untoward side) effects better.

**Fig. 1:**
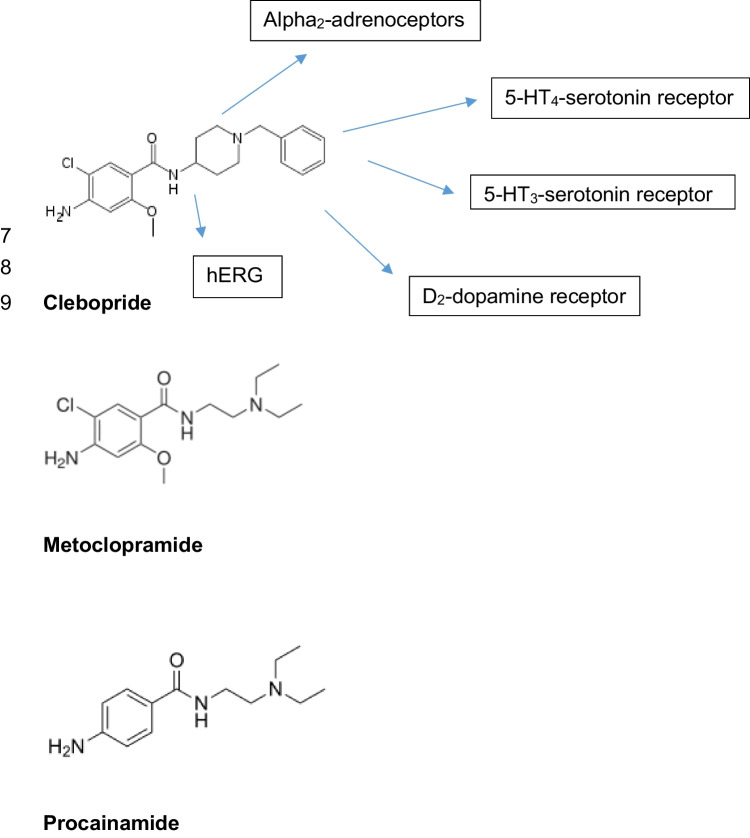
Please note how the benzene ring and its substituents were slowly made more complex, from the starting stone namely procainamide (an ancient antiarrhythmic agent) to metoclopramide and clebopride. With arrows, we indicate with which receptors clebopride is known to interact as an agonist or and antagonist or as a partial agonist. Clebopride also inhibits hERG activity, a cardiac potassium channel

Clebopride is a benzamide in its chemical structure. It was developed in a series that started with procainamide and included the gastrointestinal drug metoclopramide (Fig. 1). Like metoclopramide, clebopride acts on dopamine receptors. A sincere concern for chronic treatment with metoclopramide, a drug approved for the treatment of gastroparesis in the USA, is acute and tardive dyskinesia resulting from block of D_2_-dopamine receptors. Clebopride was developed in order to block D_2_-dopamine receptors more potently than metoclopramide. Therefore, it is not surprising that there are many clinical reports of acute dyskinesia with clebopride (e.g. Cuena Boy and Maciá Martínez 1998, Tonini et al. 2004). There are also some cardiovascular side effects reported for clebopride in patients; however, these effects were not specified (Giudicessi et al. 2018).

As far as we could find out, the possibility that clebopride might stimulate or inhibit 5-HT_4_-serotonin receptors in the heart of any species has hitherto not been explored. Previously, we demonstrated that metoclopramide is an agonist at 5-HT_4_ serotonin receptors using a mouse model with cardiac-specific overexpression of these receptors (Neumann et al. 2021a, b). In the same paper, we reported that metoclopramide was also an antagonist at 5-HT_4_ serotonin receptors in 5-HT4-TG (Neumann et al. 2021a, b). Moreover metoclopramide could exert alone positive inotropic effects (PIE) in isolated human atrial preparations (HAP; Neumann et al. 2021a, b).

Serotonin in the human exerts its PIE only via 5-HT_4_ serotonin receptors (Kaumann et al. 1990, Kaumann and Levy 2006, Neumann et al. 2023a). For instance, 5-HT_4_ serotonin receptors are functional in the human atrium and increase the force of contraction (FOC) and the current through the L-type calcium cation channels in isolated human atrial cardiomyocytes (Kaumann et al. 1990, Ouadid et al. 1992, Jahnel et al. 1993). Serotonin itself has proarrhythmic effects in the human heart as was reported decades ago (Neumann et al. 2017, Neumann et al. 2023a). This is concluded from clinical studies and from in vitro experiments in isolated human cardiac tissue (Kaumann and Sanders 1994, Kaumann and Levy 2006). Serotonin also induced arrhythmias in the atrium of mice with cardiac overexpression of the human 5-HT_4_ receptor (Keller et al. 2018). More specifically, gastrointestinal drugs that agonists at 5-HT4 receptors like cisapride or tegaserod have been removed from the market because clinical observations noted arrhythmias or coronary syndromes (Neumann et al. 2021a, b, Tack et al. 2012). However, for cisapride, the main reason for the arrhythmias seems to stem from the fact that cisapride inhibited hERG channels in the human heart which prolonged the duration of the action potential and led to torsade pointes (which can deteriorate to ventricular fibrillations, Tack et al. 2012). We noticed with cisapride in mouse atria a negative chronotropic effect and explained this by inhibition of hERG (Keller et al 2018). For this reason, we were interested in a possible negative chronotropic effect of clebopride in the mouse, if clebopride significantly inhibited the hERG channels (see Fig. 1).

Serotonin increased FOC in the failing human ventricle but not in non-failing human ventricle, possibly due to increased expression of the receptor in human heart failure (Brattelid et al. 2004). In other words, in the healthy human ventricle, serotonin alone is lacking a PIE, whereas in the healthy human atrium, serotonin increased FOC, suggesting profound regional differences in receptor densities. Of the animals studied so far, only pig and monkey hearts express functional 5-HT_4_ serotonin receptors (review: Neumann et al. 2023a). Therefore, to be independent of expensive pig or monkey models and in order to study the human receptor, we have generated and characterized transgenic mice with overexpression of functional 5-HT_4_ receptors in the heart (5-HT_4_-TG: Gergs et al. 2010, 2013, 2017). We have used this model (5-HT_4_-TG) to detect cardiac effects of 5-HT_4_ receptors of agonists or partial agonists like prucalopride, cisapride, and metoclopramide, and this motivated us to study in this model the cardiac effects of clebopride for direct comparison (Keller et al. 2018, Gergs et al. 2023). Because we basically want to understand the action of clebopride in the human heart, we used here HAP as a model system for the whole human heart.

In summary, we tested the following hypotheses:

1. Clebopride stimulates the force of contraction in isolated left atrial preparations from 5-HT_4_-TG.
2. Clebopride stimulates beating rate in spontaneously beating right atrial preparations from 5-HT_4_-TG.
3. Clebopride stimulates force of contraction in human atrial preparations via 5-HT_4_ receptors.

**Keywords: **Clebopride, 5-HT_4_-receptors, transgenic mice, human atrium

## Materials and methods

### Transgenic mice

Mice (aged about 180 days, random gender) in this study included transgenic mice (CD1 background) where the full length human 5-HT_4_-serotonin receptor is overexpressed in the heart driven by α-myosin heavy chain promoter (5-HT_4_-TG). The generation and initial characterization of these mice on a biochemical and functional levels has been reported some years ago (Gergs et al. 2010). For comparison, we used littermate wild-type animals (WT). Mice were sacrificed by cervical dislocation as demanded by our animal protection committee (permission number TM2). The mice were kept and bred in the animal facility of the medical faculty under the guidance of a veterinarian. In brief, the right or left atrial preparations from the mice were isolated and mounted under isometric conditions in organ baths as previously described (Gergs et al. 2013, Neumann et al. 1998). The bathing solution of the organ baths (10 ml volume) contained 119.8 mM NaCI, 5.4 mM KCI, 1.8 mM CaCl_2_, 1.05 mM MgCl_2_, 0.42 mM NaH_2_PO_4_, 22.6 mM NaHCO_3_, 0.05 mM Na_2_EDTA, 0.28 mM ascorbic acid, and 5.05 mM glucose. Ascorbic acid (final concentration: 0.28 mM) was used to inhibit oxidation of isoprenaline or serotonin. The solution was continuously gassed with 95% O_2_ and 5% CO_2_ and maintained at 37°C and pH 7.4 (Neumann et al. 1998). Force of contraction was quantified in electrically paced (1 Hz) isolated left atrial preparations (Grass stimulator SD9, Quincy, MA, USA). Electrical stimulation with a rectangular impulse of direct current lasted for 5 ms. The voltage was 10% higher than necessary to initiate contraction. Muscles were stretched under isometric conditions such that the maximum basal force was generated and then allowed to stabilize for 30 min before drug application started. Spontaneously beating right atrial preparations from mice were used to study any chronotropic effects. The isometric force generated (FS20 force transducer, Hugo Sachs, Freiburg, Germany) was quantified with a bridge amplifier. The signal was fed into commercial personal computers and quantified with a commercial software called LabChart 8 (AD Instruments, Oxford, England).

The drug application was in a part as follows. After equilibration of the muscles was reached, clebopride was cumulatively added to double barrelled (to keep the temperature with a water bath from Willers Laborbedarf, Münster, Germany) 10 ml organ baths (thermostat from Lauda, Lauda-Königshofen, Germany) which contained left atrial or right atrial preparations to establish concentration-response curves in the modified Tyrode’s solution. In some experiments, serotonin and isoprenaline or a serotonin receptor antagonists were added (see figure legends for details).

### Contractile studies on human preparations

The contractile studies on human preparations were done using the same setup and buffer as used in the mouse studies. In brief, force of contraction was quantified in electrically paced isolated left atrial preparations. Duration of electrical stimulation with a rectangular impulse of direct current lasted for 5 ms. The voltage was 10% higher than necessary to initiate contraction. Muscles were stretched such that the maximum basal force was generated and then allowed to stabilize for 30 min before drug application started. Basal developed force can be seen in the relevant diagrams in this paper labelled with milli Newton (mN) in the ordinates under the condition labelled control (CTR). The samples were obtained from ten male patients and four female patients, aged 45–83 years. The patients suffered from coronary diseases (two and three vessel diseases). Cardiac drug therapy included β-adrenoceptor antagonists, diuretic drugs, anticoagulants, and acetyl salicylic acid. Our methods used for atrial contraction studies in human samples have been previously published and were not altered in this study (Gergs et al. 2009, 2017). Drug addition was as described in the preceding paragraph.

### Data analysis

Data are presented as means ± standard error of the mean. Statistical significance was estimated using the analysis of variance (ANOVA) followed by Bonferroni’s post hoc test or by unpaired *t-*test, as appropriate and as indicated in the figure legends, using a commercial software (GraphPad Prism 9). A *p*-value < 0.05 was defined as significant.

### Drugs and materials

The drugs isoprenaline-hydrochloride (Merck), serotonin hydrochloride (stock solutions in water as 10 mM, Merck), and clebopride (MedChemExpress via Hycultec, Beutelsbach, Germany) at 10 mM were dissolved in dimethylsulfoxide (DMSO), serotonin, GR125487 (5-fluoro-2-methoxy-[1-[2-[(methylsulfonyl)amino]ethyl]-4-piperidinyl]-1*H*-indole-3-methylcarboxylate sulfamate (Tocris via Bio-Techne, Wiesbaden, Germany, stock solution in DMSO was 100 mM), and cilostamide (N-cyclohexyl-N-methyl-4-(1,2-dihydro-2-oxo-6-quinolyloxy)butyramide, Merck, Dreieich, Germany). All other chemicals were of the highest purity grade commercially available. Deionized water was used throughout the experiments for preparation of Tyrode’s solution. Stock solutions were prepared fresh daily.

## Results

**Fig. 2:**
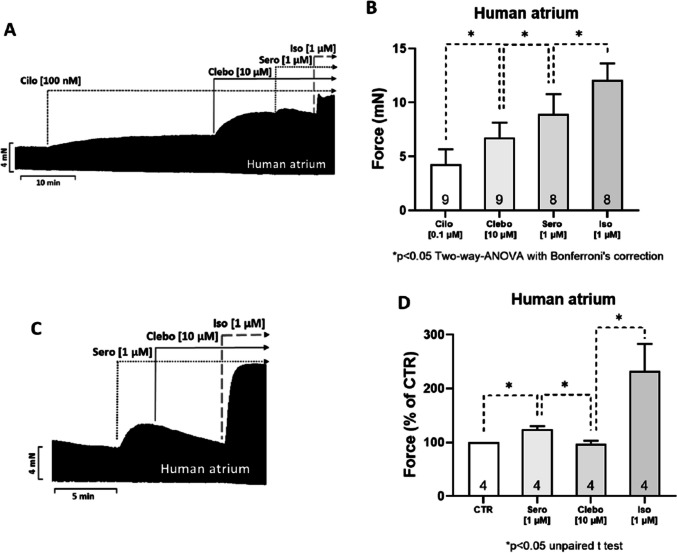
Original recording of the time-dependent positive and negative inotropic effects of clebopride on electrically stimulated human right atrial muscle strips, demonstrating its dual action as an agonist (A) or antagonist (C). To compare the efficacy of different compounds, cilostamide (Cilo, 100 nM), clebopride (Clebo, 10 µM), serotonin (Sero, 1 µM), and finally isoprenaline (Iso, 1 µM) were sequentially added to the organ bath (A). In a separate series of experiments (C), serotonin (Sero, 1 µM) was added first, followed by clebopride (Clebo, 10 µM), to evaluate its antagonistic effect. To verify the veracity of this negative inotropic effect, isoprenaline (Iso, 1 µM) was added last. In (A) and (C), horizontal lines represent time (in minutes), while vertical lines indicate the developed force of contraction (in milli Newton, mN). The summarized effects of these drugs are presented in Figure 2B and D. The ordinate axis in (B) represents the force of contraction in mN, whereas in Figure 2D, it is expressed as a percentage (%) of the pre-drug value (control, CTR). The number of experiments is indicated within the bars. * denote statistically significant differences (*p < 0.05) based on two-way ANOVA followed by Bonferroni’s multiple comparison post hoc test or, where applicable, an unpaired *t*-test between the indicated bars by the dotted lines

First, we wanted to test the effects of clebopride in the human heart. To this end, we mounted HAP in the organ bath, electrically stimulated them, and obtained concentration-response curves for clebopride on FOC. As seen in the original recording in Fig. 2A, clebopride time-dependently increased FOC, and this increase was further augmented by additionally applied serotonin. Force increased further when isoprenaline was added. These data are summarized in Fig. 2B expressed in milli Newtons (mN). Moreover, when we stimulated HAP with 1 µM serotonin and then added 10 µM clebopride, we detected a negative inotropic effect. This is depicted in an original recording (Fig. 2C), and several such data are summarized in Fig. 2D.

**Figure 3:**
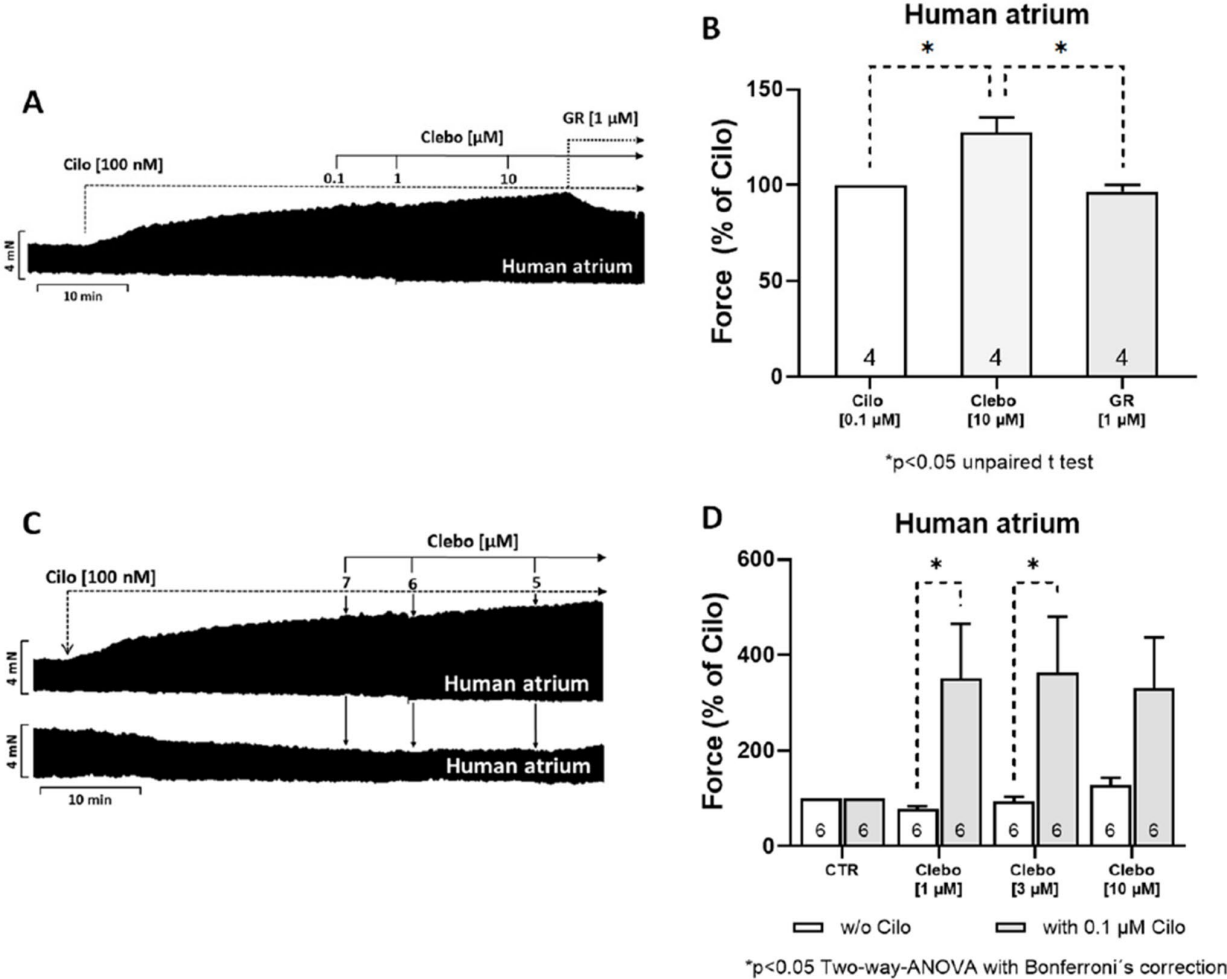
**A** Original recording of the time-dependent positive inotropic effect of clebopride (measured in milli Newton, mN) in electrically stimulated human right atrial muscle strips. Cilostamide (Cilo, 100 nM) was added first, followed by increasing concentrations of clebopride (Clebo, 0.1–10 µM), and finally the 5-HT₄ serotonin receptor antagonist GR125487 (GR, 1 µM). These data are summarized in the bar diagram in **B**. Moreover, clebopride (Clebo, 0.1–10 µM) was applied either alone (**C**, bottom) or in combination with cilostamide (Cilo, 100 nM; **C**, top). The data from experiments such as those shown in **C** are summarized in **D**, indicating that clebopride alone does not increase contractile force but does so in the presence of the phosphodiesterase III inhibitor cilostamide. In **A** and **C**, horizontal lines represent time (in minutes), while vertical lines indicate developed force of contraction (in mN). In **B** and **D**, the ordinate axis represents the force of contraction as a percentage of the pre-drug value (Cilo, 100 nM). The number of experiments is indicated within the bars. * denote statistically significant differences (*p < 0.05) based on two-way ANOVA followed by Bonferroni’s multiple comparison post hoc test or, where applicable, an unpaired *t*-test between the indicated bars by the dotted lines

Cumulatively applied clebopride in the presence of cilostamide (an inhibitor of phosphodiesterase 3, the main phosphodiesterase of the human heart (Kamel et al. 2023), used by us and others before (Christ et al. 2006, Gergs et al. 2023)) increased at each applied concentration the FOC. This effect is illustrated in an original recording (Fig. 3A) and quantified in Fig. 3B. Finally, we noticed that 1 µM GR12457 (an antagonist at 5-HT4 receptors) reduced FOC in HAP after clebopride administration (Fig. 3A and B). Cilostamide was not given in mouse studies because cilostamide does not increase force of contraction in mouse atria (Neumann et al. 2019). This is explained by the observation that not phosphodiesterase 3 but phosphodiesterase 4 is relevant in the mouse heart (Kamel et al. 2023).

Please note, we only recorded a PIE to clebopride in the presence of cilostamide (Fig. 3C, top), but not in its absence (Fig. 3C, bottom). Several similar experiments were quantified in Fig. 3D. Under these experimental conditions, we analysed additional time parameters that might be predicted to be altered if the 5-HT4 receptors were involved in signal transduction. In line with our previous publications, we observed that cilostamide reduced time to peak tension. Additionally applied clebopride, serotonin or isoprenaline (see Fig. 2A) did not reduce time of relaxation significantly further, while mean values tended to decrease.

**Fig. 4:**
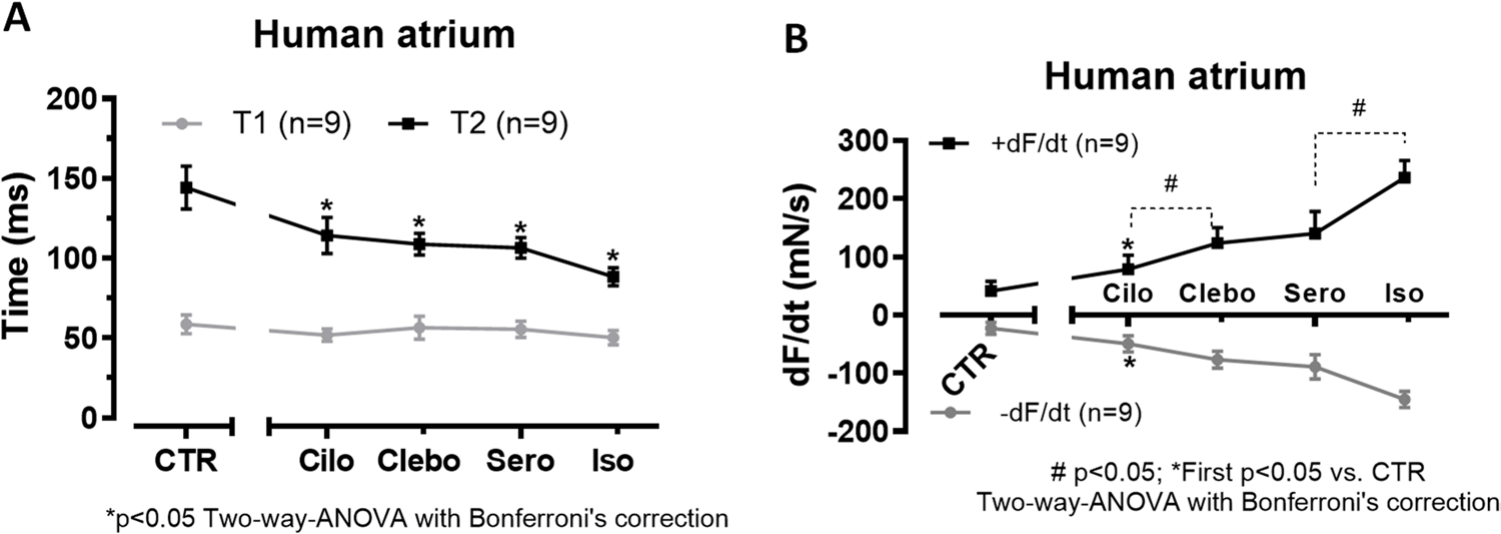
In samples such as those shown in Figure 2, we measured the effects of clebopride on contractile parameters. Cilostamide (Cilo, 100 nM) was administered first, followed by clebopride (Clebo, 10 µM), serotonin (Sero, 1 µM), and finally isoprenaline (Iso, 1 µM; Figure 2A). Data on time to peak tension (T1) and time of relaxation (T2) in ms are presented in **A**, while data on the rate of tension development (+dF/dt) and rate of relaxation (-dF/dt) in milli Newtons per second (mN/s) under the same conditions are shown in **B**. “*n*” indicates number of experiments. Asterisks (*) indicate significant differences (*p* < 0.05) compared to pre-drug values (CTR). # denote significant differences (*p* < 0.05) between the values indicated by the dotted lines. Statistical analysis was performed using two-way ANOVA followed by Bonferroni’s multiple comparison post hoc test

As concerns time to peak tension (T1 in Fig. 4A), cilostamide—but not additional clebopride or serotonin—could reduce time to peak tension. Under these conditions, cilostamide increased rate of tension development (slope max) and rate of tension relaxation (slope min) in Fig. 4B. Additionally applied clebopride and serotonin further increased the mean values of these parameters (Fig. 4B).

**Fig. 5:**
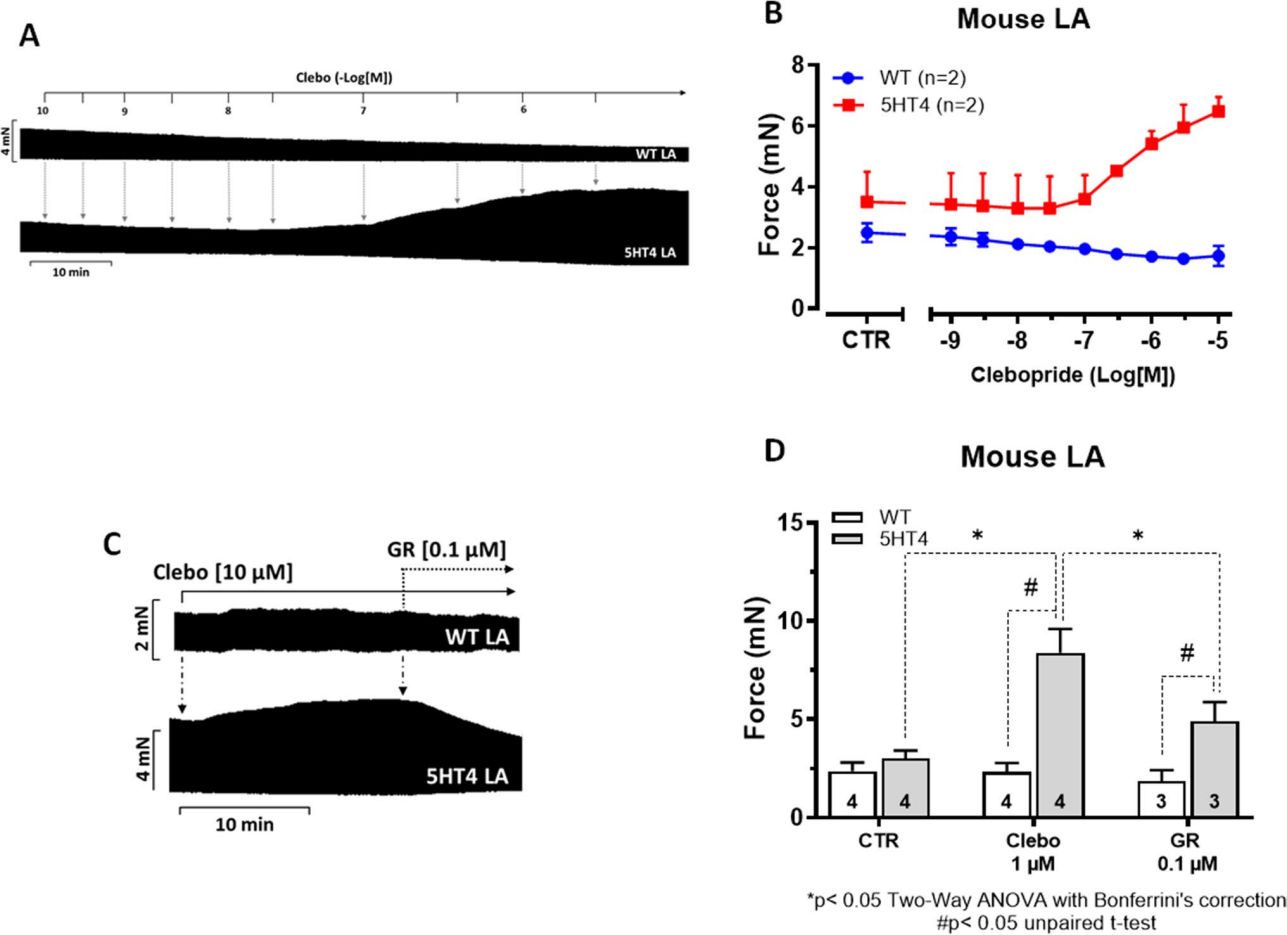
Original recordings of isolated, electrically stimulated mouse left atrial preparations (LA) from 5-HT₄-TG (lower trace) and WT (upper trace) are shown in **A**. Clebopride, administered cumulatively from 0.1 nM to 10 µM, induced a time- and concentration-dependent positive inotropic effect in 5-HT₄-TG. **B** illustrates that the positive inotropic effect of clebopride is selectively mediated via the 5-HT₄ receptor, occurring only in TG (age: 151.5 ± 68.3 days; ♀: 4) and not in WT (age: 97.8 ± 4 days; ♂: 4). The vertical axis represents the contraction force in milli Newtons (mN), while the horizontal axis indicates clebopride concentration in negative decadic molar values (-Log [M]). **C** (original recording) shows that the positive inotropic effect of clebopride is antagonized by GR125487 (GR, 0.1 µM). Several datasets similar to those in Figure 2C are summarized in **D**, demonstrating that clebopride (Clebo, 1 µM) induces a time- and concentration-dependent positive inotropic effect in 5-HT₄-TG, which is effectively antagonized by GR125487 (GR, 0.1 µM). The number of experiments conducted is indicated within the bars of the graph. * denote significant differences (*p* < 0.05) compared to the values indicated by the dotted lines, as determined by a two-way ANOVA followed by Bonferroni’s multiple comparison post hoc test. # indicate a *p*-value < 0.05 between the values represented by the dotted lines, as analysed using an unpaired *t*-test

To corroborate our findings in human atrium and to study the effects on beating rate, we next studied a well-characterized transgenic mouse model for the cardiac function of the human 5-HT_4_-serotonin receptor. More specifically, we had expressed already the 5-HT_4_-serotonin receptor in the heart using a cardiomyocyte specific promoter (Gergs et al. 2010, 2013). In atria from these animals—but not in atria from WT—serotonin exerted positive inotropic and a positive chronotropic effect (left atrium versus right atrium, respectively, Gergs et al. 2013). As seen in an original recording (Fig. 5A), clebopride induced a time- and concentration-dependent PIE only in LA from 5-HT_4_-TG (Fig. 5A, bottom), but not in WT (Fig. 5A top). This is summarized in Fig. 5B. Moreover, the PIE of clebopride in LA from 5-HT_4_-TG could be almost completely reversed by the 5-HT_4_-serotonin receptor antagonist GR125487 (Fig. 5C). Such data are summarized in Fig. 5D.

**Fig. 6:**
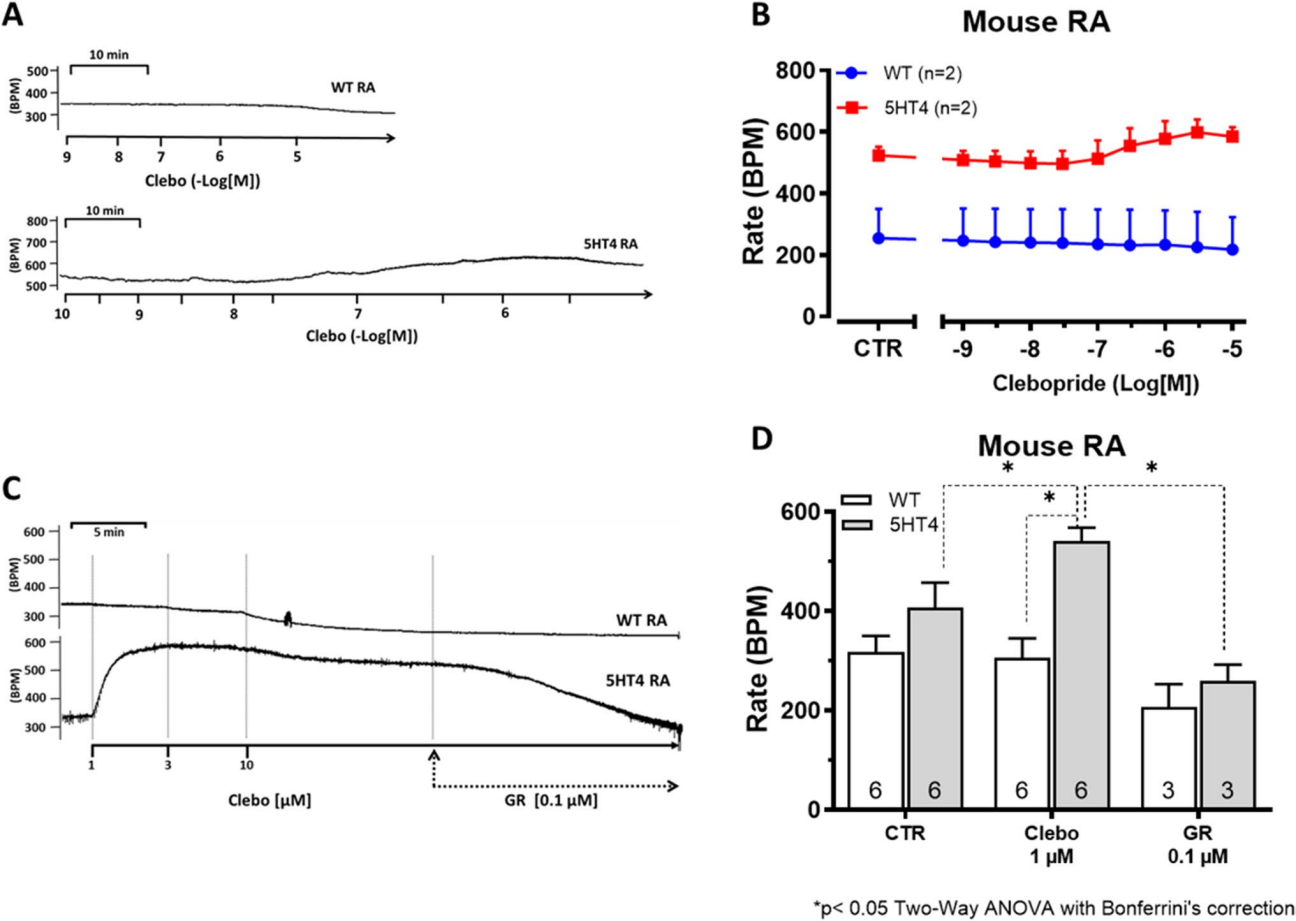
Original recordings of the effects of clebopride on right atrial (RA) preparations from 5-HT₄-TG (lower trace) and WT (upper trace) are shown in **A**. Clebopride induced a time- and concentration-dependent positive chronotropic effect in isolated, spontaneously beating RA preparations from 5-HT₄-TG, but not in RA from WT. This is summarized in **B**. In **A** and **B**, the ordinates represent beating rates in beats per minute (BPM), and the horizontal lines indicate clebopride concentrations in negative decadic molar units (-Log [M]). The positive chronotropic effects of clebopride are antagonized by the 5-HT₄ receptor antagonist GR125487 (GR; 0.1 µM), as shown in the original recordings in **C**. The summarized data from these experiments are presented in **D**. It is important to note that data were collected from 5-HT₄-TG (age: 212.5 ± 109.7 days; ♀: 6), and, for direct comparison, data from WT (age: 148.3 ± 115.3 days; ♂: 5, ♀: 1) were also included. The number of experiments conducted is indicated within the bars of the graph. The ordinates represent beats per minute (BPM). The abscissa in **C** shows clebopride concentrations in negative decadic molar units, whereas **D** presents the drug concentrations (CTR, pre-drug value; Clebo, clebopride, 1 µM; GR, GR125487, 0.1 µM) as a bar graph. Asterisks (*) denote significant differences (*p* < 0.05) compared to the values indicated by the dotted lines, as determined by a two-way ANOVA followed by Bonferroni’s multiple comparison post hoc test

Clebopride also increased beating rate in RA from 5-HT_4_-TG (Fig. 6A, bottom), but not in WT (Fig. 6A, top). This is summarized in Fig. 6B. Moreover, we applied in RA from 5-HT_4_-TG, initially clebopride in order to elevate the beating rate (beats per minute, BPM) and then applied the antagonist GR 125487 which reduced the elevated beating rate (Fig. 6C, original recordings). These findings are summarized for 1 µM clebopride in Fig. 6D.

**Fig. 7:**
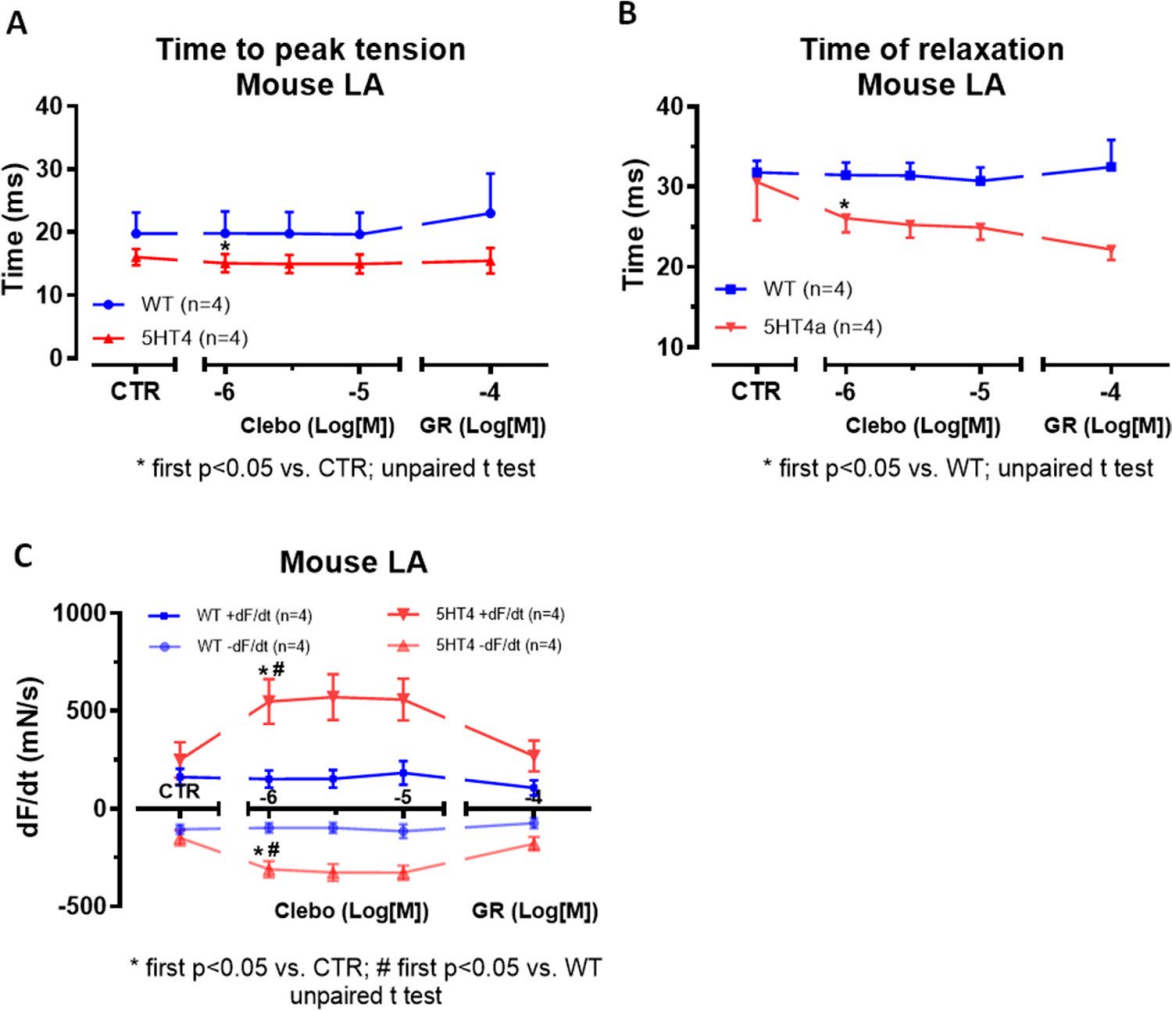
As shown in Figure 5, the positive inotropic effect of clebopride (Clebo, 1–10 µM) occurs exclusively in left atrial (LA) preparations from 5-HT₄-TG (age: 151.5 ± 68.3 days; ♀: 4), whereas this effect is absent in WT (age: 97.8 ± 4 days; ♂: 4). The 5-HT₄ receptor antagonist GR125487 (GR, 0.1 µM) abolishes this increase in contractile force. Data on time to peak tension (T1) and relaxation time (T2) in ms are presented in **A** and **B**, respectively, while data on the rate of tension development (+dF/dt) and relaxation (-dF/dt) in milli Newtons per second (mN/s) under the same conditions are shown in **C**. The number of experiments is indicated in parentheses followed by “*n*” in each case

Finally, we were interested in the effects of clebopride on contractile parameters on time of contraction in LA from 5-HT_4_-TG to compare this with our data in HAP. It turned out that clebopride reduced time to peak tension (Fig. 7A) and time of relaxation (Fig. 7B). This is expected in agents that increase phosphorylation of phospholamban via the 5-HT_4_-serotonin receptor (Gergs et al. 2013). Furthermore, the rate of tension development and the rate of relaxation were augmented in absolute values by clebopride (Fig 7C).

## Discussion

### Main new finding

The main new finding is a follows: clebopride acts as a partial agonist via 5-HT_4_ receptors in the beating human heart.

### Mechanism of clebopride

Clebopride was invented to improve the clinical usefulness of metoclopramide. It was felt that metoclopramide was suboptimal for gastrointestinal mobility diseased (Ramirez and Richter 1993). Clebopride was designed to increase the anti-dopaminergic activity of metoclopramide, and this is hypothesized to improve the effect of clebopride compared to metoclopramide on gastrointestinal mobility (Ramirez and Richter 1993). The amine side chain of metoclopramide was replaced in clebopride by an N-substituted piperidine nucleus (Ramirez and Richter 1993, Fig. 1). Indeed, clebopride is a potent antagonist at D_2_-dopamine receptors (K_i_-value about 2 nM, Tonini et al. 2004), but it also acts on 5-HT_3_ receptors and at α_2_-adrenoceptors (Takeda et al. 1991). In addition, clebopride poorly binds to D_1_-dopamine receptors but binds with high affinity to D_4_-dopamine receptors and with intermediate affinity also to D_3_-dopamine receptors in radioactive ligand binding assays (Einsiedel et al. 2003). However, these antagonisms at these receptors are not known to possibly increase force of contraction in the human heart. Hence, an interaction of clebopride with these receptors cannot explain the PIE of clebopride in HAP, and these receptors fell from consideration.

The only previous report on cardiac action of clebopride was the following electrophysiological study: in human ether-à-go-go-related gene (hERG)-stably transfected Chinese hamster ovarian cells, clebopride inhibited the hERG channel with an IC_50_-value of 0.6 µM (Kim et al. 2007). In addition, 10 µM clebopride prolonged the duration of the action potential in rabbit Purkinje fibres (Kim et al. 2007). This prolongation could contribute, at least in part, to PIE of clebopride in HAP: if the action potential is longer, more time is available for calcium ions to stream in via L-type calcium channels (LTCC). As a consequence, more calcium ions are in the cytosol, where they could interact with myofilaments to initiate contraction. Of some concern, the inhibition of potassium channels might lead to arrhythmias due to clebopride.

However, we believe that clebopride increases FOC and beating rate mainly as an agonist at cardiac human 5-HT_4_-serotonin receptors: This is because clebopride increases contractility only in atria from 5-HT_4_-TG and not in WT. By comparing the concentration-response curves of clebopride to that of serotonin in atrial preparations (human atrium (Gergs et al. 2009), mouse atrium in 5-HT_4_-TG (Gergs et al. 2013), we can conclude that clebopride may act as a full agonist at 5-HT_4_-serotonin receptors in left and right atrium. However, these are experiments which were performed on different patients (Gergs et al. 2009) and not the same patients studied here which is a limitation of this study.

Our findings in HAP are in line with our animal studies: first of all, clebopride increased FOC in HAP. Clebopride activates receptors in the human heart, here the human atrium. These receptors are functionally blocked by a 5-HT_4_ serotonin receptors antagonist, as this antagonist reduced FOC in HAP. This would also argue that the PIE of clebopride is not due to an inhibition of potassium channels (hERG), as such an effect should not be blocked by GR125487.

As concerns, human atrium, clebopride alone does not increase FOC. This is shared with other similar 5-HT4 receptor ligands like bromopride and similar drugs like mosapride or zacopride (Rayo Abella et al. 2025, Neumann et al. 2024a, Neumann et al. 2024b). When we pretreated the HAP with a low concentration of cilostamide, then we detected a PIE to clebopride. We chose cilostamide because it inhibits quite selectively phosphodiesterase III, the main isoenzyme in the human heart (Kamel et al. 2023). The concentration of cilostamide was chosen in order not to increase FOC maximally so that other compounds like mosapride (Neumann et al. 2024b) or bromopride (Rayo Abella et al. 2025) or clebopride (this study) were still able to increase FOC further. We suggest that cilostamide inhibits rapid degradation of cAMP that was formed when clebopride stimulated the 5-HT4 receptor and enhanced the formation of cAMP via GTP binding proteins and adenylyl cyclase (Neumann et al. 2023a for review).

We furthermore show that clebopride can antagonize the PIE of 1 µM serotonin in HAP and LA. Thus, clebopride behaves as both an agonist and an antagonist at 5-HT_4_ serotonin receptors in the human heart.

### Species differences

Of note, clebopride acted more potently to raise FOC in TG than in human atrium. This is in line with our previous work on bromopride, cisapride, prucalopride, mosapride, zacopride, tegaserod, or metoclopramide (Keller et al. 2018, Neumann et al 2024a, Neumann et al. 2024b, Hesse et al. 2024, Rayo Abella et al. 2025). The expression of 5-HT_4_ serotonin receptors in mouse hearts from 5-HT4-TG was higher in comparison to human hearts. We have previously described this overexpression on mRNA level (Gergs et al. 2010, Neumann et al. 2021b). We would argue that the 5-HT_4_-TG offers the possibility of amplifying any effect of agonists at 5-HT_4_ serotonin receptors. One can argue that if a putative agonist does not act in 5-HT_4_-TG, this agonist is unlikely to work in human cardiac tissue as an agonist at 5-HT_4_ receptors.

### Effects on beating rate

Next, we would like to discuss also our findings in RA. We assume that, like 5-HT, clebopride also stimulated 5-HT_4_ serotonin receptors in the mouse heart. This conclusion is based on the observation that the positive chronotropic effects of clebopride are absent in RA from WT and present in 5-HT_4_-TG. Moreover, the positive chronotropic effects of clebopride are antagonized by GR125487, a 5-HT_4_ serotonin receptors antagonist. Clebopride acted unlike various other agonists in our hands (cisapride, prucalopride, metoclopramide). It is well established that a positive chronotropic effect of serotonin in the human heart is mediated by 5-HT_4_-serotonin receptors (Neumann et al. 2023a). Hence, we expect that clebopride might induce a tachycardia in patients via this mechanism. But this needs to be confirmed by a clinical trial.

### Clinical relevance

Interestingly, seems to be in some countries of clinical relevance. For instance, when cisapride was pulled from the market for fear of arrhythmias (Tonini et al. 2004), clebopride was more often prescribed in Spain and Italy. Now one can ask how high concentrations of clebopride such as 10 µM (this study) could possibly be reached in patients. Now, clebopride is metabolized via the cytochrome CYP3A4. Hence, inhibitors of CYP3A4, like fluconazole or grapefruit juice, would inhibit the metabolism of clebopride and might thus increase its plasma concentration (Sun and Scott 2011).

Hence, we can predict that clebopride can, in principle, stimulate human atrial 5-HT_4_ serotonin receptors. This stimulation might lead to a PIE which should be beneficial. This effect should be limited to the left and right atrium, and this will have only a limited contribution to the cardiac performance. Indeed, the non-failing heart, serotonin was ineffective to raise FOC in the human ventricle (Brattelid et al. 2004). However, in heart failure patients, the density of 5-HT_4_ serotonin receptors (as measured at the mRNA level) was increased, and this may explain that in their ventricular muscle preparations, serotonin was able to increase FOC (Brattelid et al. 2004). Therefore, clebopride might increase ventricular FOC in heart failure patients. A caveat is in order: stimulation of 5-HT_4_ serotonin receptors in the human heart can lead to arrhythmias, and as such would be detrimental (Kaumann and Levy 2006). Such arrhythmias could be treated in theory by approved receptor antagonists (tropisetron, piboserod (Kaumann et al. 1990, Kjekshus et al. 2009)). However, this would simultaneously block serotonin receptors in the gut, and hence, any intestinal benefit of clebopride may well be missing.

### Further limitations of the study

All drugs were always given in the same order, without either changing the order with washouts in between or with time-matched controls. However, this was done to facilitate comparison with our earlier work where we used the same procedure (Keller et al. 2018, Neumann et al. 2021a). Moreover, we only observed effects of clebopride in the presence of the phosphodiesterase inhibitor cilostamide. Cilostamide is not used in clinical practice. However, at least the phosphodiesterase inhibitors, pimobendan and levosimendan are used in the clinic (von der Leyen et al. 1991, Orstavik et al. 2014, Abella et al. 2023). Perhaps, when theophylline (which is an unspecific broad phosphodiesterase inhibitor) is used at high dosage, a relevant inhibition of phosphodiesterase 3 might also occur (Barnes 2013, Spina and Page 2017). Hence, there are clinical scenarios where clebopride might stimulate the force of contraction in the human atrium because the patient already takes a phosphodiesterase 3 inhibitor. In addition, it is conceivable that effects of clebopride on central D_2_-dopamine receptors and an inhibitory action on hERG may also contribute to arrhythmias under the therapy with clebopride (Vos 2008, Giudicessi et al. 2018, Neumann et al. 2023b). The mechanism(s) of any arrhythmias by clebopride need to be elucidated in subsequent clinical studies.

In summary, we can now address the hypotheses raised in the “Introduction” in this way: clebopride raised force of contraction and beating rate in 5-HT_4_-TG not WT and elevated force of contraction as a partial agonist in the human atrium via 5-HT_4_ receptors.

## Data Availability

The data of this study are available from the corresponding author upon reasonable request.
